# Diffusion tensor imaging of *post mortem* multiple sclerosis brain

**DOI:** 10.1016/j.neuroimage.2006.12.010

**Published:** 2007-04-01

**Authors:** Klaus Schmierer, Claudia A.M. Wheeler-Kingshott, Phil A. Boulby, Francesco Scaravilli, Daniel R. Altmann, Gareth J. Barker, Paul S. Tofts, David H. Miller

**Affiliations:** a Institute of Neurology, University College London, NMR Research Unit, Box 117, Queen Square, London WC1N 3BG, UK; b Department of Molecular Neuroscience, Division of Neuropathology, Institute of Neurology, University College London, UK; c London School of Hygiene and Tropical Medicine, University of London, UK; d King’s College London, Institute of Psychiatry, Department of Clinical Neuroscience, Centre for Neuroimaging Sciences, London, UK

## Abstract

Magnetic resonance imaging (MRI) is being used to probe the central nervous system (CNS) of patients with multiple sclerosis (MS), a chronic demyelinating disease. Conventional *T*_2_-weighted MRI (cMRI) largely fails to predict the degree of patients' disability. This shortcoming may be due to poor specificity of cMRI for clinically relevant pathology. Diffusion tensor imaging (DTI) has shown promise to be more specific for MS pathology. In this study we investigated the association between histological indices of myelin content, axonal count and gliosis, and two measures of DTI (mean diffusivity [MD] and fractional anisotropy [FA]), in unfixed *post mortem* MS brain using a 1.5-T MR system. Both MD and FA were significantly lower in *post mortem* MS brain compared to published data acquired *in vivo*. However, the differences of MD and FA described *in vivo* between white matter lesions (WMLs) and normal-appearing white matter (NAWM) were retained in this study of *post mortem* brain: average MD in WMLs was 0.35 × 10^− 3^ mm^2^/s (SD, 0.09) versus 0.22 (0.04) in NAWM; FA was 0.22 (0.06) in WMLs versus 0.38 (0.13) in NAWM. Correlations were detected between myelin content (Tr_myelin_) and (i) FA (*r* = − 0.79, *p* < 0.001), (ii) MD (*r* = 0.68, *p* < 0.001), and (iii) axonal count (*r* = − 0.81, *p* < 0.001). Multiple regression suggested that these correlations largely explain the apparent association of axonal count with (i) FA (*r* = 0.70, *p* < 0.001) and (ii) MD (*r* = − 0.66, *p* < 0.001). In conclusion, this study suggests that FA and MD are affected by myelin content and – to a lesser degree – axonal count in *post mortem* MS brain.

## Introduction

Histopathologically, central nervous system (CNS) tissue of individuals with multiple sclerosis (MS) is characterized by demyelination, axonal damage, gliosis, inflammation and often some degree of remyelination. Whereas microscopically these features can be readily appreciated, it has been proven difficult to distinguish them *in vivo* using neuroimaging methods. *T*_2_ weighted (*T*_2_w) and gadolinium (Gd)-enhanced *T*_1_-weighted (*T*_1_w; both techniques often summarized as “conventional”) magnetic resonance imaging (MRI) displays MS white matter (WM) lesions (WMLs) with high sensitivity and is helpful in diagnosis (McDonald et al., 2001; Polman et al., 2005). Aside from a relationship between Gd enhancement and inflammation (Hawkins et al., 1990; Katz et al., 1993), however, such conventional MRI (cMRI) techniques do not distinguish other histopathological substrates of MS (Moore, 2003). The inability of cMRI to reflect MS pathology in more detail may be one of the major reasons why the correlation between cMRI measures and disability is only modest (Kappos et al., 1999; Brex et al., 2002). Complementing cMRI, quantitative MR measures have been developed that are potentially more pathologically specific. These include diffusion-weighted MRI (DWI), volumetric measurements, magnetization transfer (MT) imaging, quantitative *T*_1_- and *T*_2_-relaxation time (RT) measurements, and MR spectroscopic metabolite concentrations (Tofts, 2003).

Increasing pathological specificity is worthwhile as it may help to (i) further elucidate the pathogenesis of MS and (ii) monitor treatments aimed at modifying specific processes. The investigation of *post mortem* tissue allows the direct assessment of the relationship between MR measures and underlying pathology. Such experiments have so far provided insights into the pathological correlates of several MR indices including *T*_1_ hypo-intensity (van Walderveen et al., 1998; van Waesberghe et al., 1999; Barkhof et al., 2003), *T*_1_- (Schmierer et al., 2004a) and *T*_2_-RT (Moore et al., 2000) and MT ratio (van Waesberghe et al., 1999; Barkhof et al., 2003; Schmierer et al., 2004a).

DWI relies on the detection of changes in the random translational motion of water molecules in tissue. For a comprehensive description of the motion of water molecules, the diffusion tensor can be estimated, provided diffusion tensor MRI (DTI) is employed (Basser et al., 1994; Le Bihan et al., 2001; Bammer et al., 2005). DTI requires the measurement of the MR signal with diffusion sensitization along at least six non-colinear non-coplanar directions and allows determination of three mutually perpendicular eigenvectors (indicating the direction of the diffusion) and the corresponding eigenvalues (giving its magnitude). Various mathematical combinations of these values provide rotationally invariant diffusion indices (i.e., measurements that characterize a specific voxel independently of positioning in the scanner) including mean diffusivity (MD) and fractional anisotropy (FA). The latter describes the fact that in CNS WM, water molecules are more likely to diffuse parallel to, rather than across, fiber tracts. The FA value increases with this anisotropy and provides a spatial depiction of anisotropic areas, whereas MD is an index of the magnitude of water diffusion regardless of its direction (Wheeler-Kingshott et al., 2003a).

DWI is sensitive to pathological processes that alter tissue integrity, as these may result in changes to (i) the number and permeability of biological barriers that normally restrict the motion of water molecules, (ii) the relative sizes of intra-cellular, extra-cellular or other compartments, or (iii) other tissue properties affecting tissue anisotropy and diffusivity.

Using experimental allergic encephalitis (EAE), an animal model of MS, an increase in diffusivity in MR detectable lesions has been shown to be associated with a number of tissue alterations including fibrosis of vessel walls, interstitial edema, vacuolization, vesicular degeneration, myelin damage (Verhoye et al., 1996), inflammatory infiltrates (Richards et al., 1995) and blood–brain barrier leakage (Broom et al., 2005). However, reduced diffusivity has also been observed in EAE, particularly in the lesion center with abundant astrocytosis and densely packed macrophages (Broom et al., 2005).

DWI has been widely applied to probe MS *in vivo* (Horsfield and Jones, 2002; Filippi et al., 2003; Symms et al., 2004; Rovaris et al., 2005; Bammer et al., 2005; Ge et al., 2005). Recently, it has been suggested that DTI metrics provide a rather specific measure of axonal loss (Hesseltine et al., 2006). However, investigation of the pathological correlates of DWI in MS has been scarce (Mottershead et al., 2003; Wheeler-Kingshott et al., 2003b; Schmierer et al., 2004b; Fox et al., 2005). In this study we aimed to systematically explore the association between quantitative histological measures of myelin content, axonal count and gliosis and two important indices of DTI (MD and FA) in *post mortem* brain samples of 16 patients with MS.

## Material and methods

### Patients/samples

This study was approved by the Joint Ethics Committees of The National Hospital for Neurology and Neurosurgery and the Institute of Neurology, UCL. *Post mortem* brain slices of 16 patients with MS (15 women and 1 man) were provided by the UK Multiple Sclerosis Tissue Bank (MSTB) based at Charing Cross Hospital, Imperial College School of Medicine, London, UK. The mean age of the patients was 59 years (SD: 13 years; range: 34–82); their mean disease duration was 25 years (9; 6–43). The course of MS (Lublin and Reingold, 1996) and the disability of the patients were assessed retrospectively from the case records collected at the MSTB. Disability was estimated using the expanded disability status score (EDSS) scale (Kurtzke, 1983). The brain weight and the pH of the cerebro-spinal fluid (CSF) of each patient were provided by the MSTB.

Brains were retrieved by the MSTB a mean of 16 h (SD: 6 h; range: 7–28 h) after death. One coronal brain slice (thickness: 1 cm) of one hemisphere in each case was used for this study. The mean time between tissue retrieval and dissection of the brain slices was 4 h (SD: 2; median: 4; range: 1–8); the mean time between death and MR scanning was 46 h (SD: 24; median: 39; range: 10–107). The brain slices were stored in sealed plastic bags at 2–8 °C until 3 h before scanning when they were taken out of the refrigerator and plastic bags, wrapped in PVC foil to minimize dehydration and left to reach scanner room temperature. To control for the effects of temperature on the results, the temperature of the samples was assessed immediately after scanning using a K-type hypodermic thermocouple temperature probe connected to a HI 93551 thermometer (Hanna Instruments Ltd., Leighton Buzzard, UK). After measuring the temperature specimens were immersed in 10% buffered formalin solution.

### MRI

Scanning was performed on a GE Signa Horizon Echospeed 1.5-T MR system (General Electric, Milwaukee, WI, USA). All MR sequences were acquired as a single slice (thickness: 5 mm), which was centered parallel to the coronal surface of the specimen.

The following sequences were performed:

(i) 2D spin echo (SE) *T*_1_w; repetition time [TR] 540 ms; echo time [TE] 18 ms; and dual SE proton density (PD), and *T*_2_w (TR 2000; TE 30 and 120 ms; flip angle 90°, matrix size 256 × 256). All these sequences were acquired twice, once with a field of view [FOV] of 300 × 300 mm^2^ (to allow visualization of fiducial markers of the custom-built sample support, see further below), and again with a FOV of 240 mm × 180 mm giving better resolution of anatomical structures and lesions.
(ii) multi-shot diffusion-weighted spin echo EPI (TR/TE: 3000 ms/86 ms). The gradient *b* factor was 1940 s/mm^2^, number of shots = 8, number of excitations = 4 (acquired separately), FOV = 12 × 12 cm^2^, matrix 48 × 48 (reconstructed as 64 × 64) resulting in a pixel size of 2.5 × 2.5 mm^2^ (reconstructed 1.9 × 1.9 mm^2^). For each shot and average, six diffusion-weighted images along six non-colinear directions, and one non-diffusion-weighted (*b* = 0) image (i.e., with *b* = 0) were acquired. The directions of the diffusion weighting were selected to apply maximum gradient strength along two axes at a time and to obtain a high *b* factor without increasing TE or loosing signal-to-noise. The scheme used was (0 0 0, 1 1 0, 1 0 1, 0 1 1, − 1 1 0, − 1 0 1, 0 − 1 1). Data were processed to determine the diffusion tensor on a pixel-by-pixel basis on each slice. Magnitude images of each slice were reconstructed and averaged off-line, prior to being processed to determine the diffusion tensor on a pixel-by-pixel basis. MD and FA were calculated from the principal diffusivities of the diffusion tensor (Wheeler-Kingshott et al., 2003b). Note that high *b* values (reducing signal-to-noise) were necessary because of the different range of the apparent diffusion coefficient in *post mortem* brain.

All scans and maps were displayed on a Sun workstation (Sun Microsystems, Mountain View, CA, USA) using DispImage (Plummer, 1992). Regions of interest (ROIs) were defined and labeled on the *T*_2_w scans, as follows: (i) areas of hyper-intense signal suspected to be MS WMLs and (ii) two to three regions of normal-appearing white matter (NAWM). ROIs on *T*_2_w scans were inherently registered with their respective *T*_1_w images. Due to the difference in geometrical distortions, however, the position of the ROIs on *b* = 0 scans had to be determined visually with reference to the corresponding *T*_2_w images. The ROIs on *b* = 0 images were then marked and co-registered with the MD and FA maps.

In order to optimize correspondence between ROIs defined on MRI with their pathological substrate in the specimens a stereotactic procedure (StP) was employed (Schmierer et al., 2003). *T*_1_w, *T*_2_w, and PD images acquired with a FOV of 300 × 300 mm^2^ were used to determine the co-ordinates of (i) the ROIs (with reference to the images acquired with a FOV of 240 mm × 180 mm) and (ii) the fiducial points of the localizer of the stereotactic system. The co-ordinates obtained at the scanner were then processed using the StereoCalc™ program (Radionics, Burlington, MA, USA) mounted on a laptop computer to give the co-ordinates that allowed – in the dissection theatre – the pointer of a Cosman–Roberts–Wells arc system (Radionics, Burlington, MA, USA) to be guided towards the target points (i.e., the ROIs) in the specimen. Every target point was then marked with a small dot of tissue dye on the surface of the specimen. In nine/16 specimens the entire MR and StP protocol was performed in one session before fixation of the tissue. In seven/16 cases StP had to be performed following a separate MR session after the tissue had been fixed as previously described (Schmierer et al., 2003).

### Pathological and morphometric procedures

**Fig. 1:**
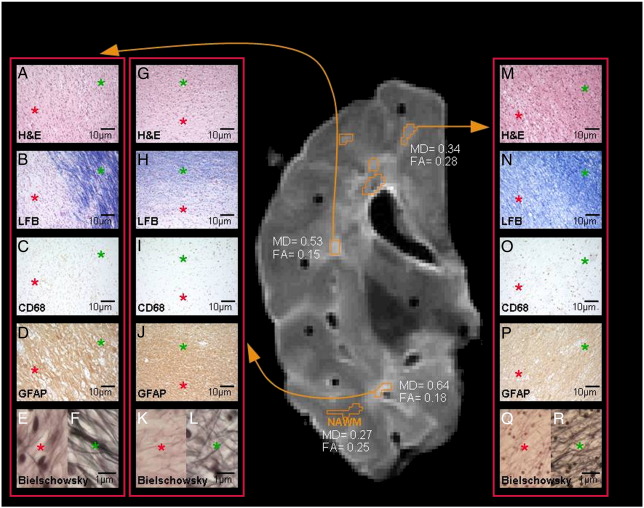
Correlation of MRI and histopathology in *post mortem* multiple sclerosis brain (same case as in Fig. 2). On the *T*_2_-weighted scan of a coronal brain slice seven exemplary regions of interest (marked in orange) were identified as either normal-appearing white matter (NAWM) or white matter lesions (WMLs). Three WMLs are matched to respective histopathological sections, which were stained for hematoxylin and eosin (H&E), Luxol fast blue (LFB), CD68, glial fibrillary acid protein (GFAP) and Bielschowsky silver impregnation. Sections A–F illustrate a demyelinated WML with moderate infiltration by CD68-positive cells indicating chronic inflammatory activity (chronic active WML), and an axonal loss of 74% (compared to NAWM). Sections G–L show a hypo-cellular demyelinated lesion with very little inflammatory activity (chronic inactive WML). Axonal loss in this WML was 93%. Sections M–R show a remyelinated WML again with very little inflammatory activity (remyelinated WML) and an axonal loss of only 42%. Due to their high magnification (× 1250) images of Bielschowsky stained sections were divided into two halves (WML on the left and NAWM on the right). All other sections cover WMLs (red asterisks) as well as NAWM (green asterisks). MD = mean diffusivity × 10^− 3^ [mm^2^/s]; FA = fractional anisotropy.

Tissue blocks of approximately 1.5 cm × 1.5 cm × 1 cm in volume and centered on each ROI were dissected. The blocks were then cut in half using a 5 mm deep iron angle resulting in two blocks of equal thickness with the cut surface corresponding to the center of the MR imaging plane. All dissected blocks were marked with notches at known positions, usually on the ventral and lateral cut surfaces, in order to assure the orientation in space after further processing. Blocks were processed for embedding in paraffin. Sections were stained with hematoxylin and eosin (H and E), Luxol fast blue (LFB), and Bielschowsky’s silver impregnation. Immunocytochemistry included antibodies to glial fibrillary acidic protein (GFAP, 1/1500) and CD68 (1/100; both from Dako, Glostrup, Denmark) Fig. 1.

Histologically, MS WMLs were defined in comparison to surrounding NAWM as clearly distinct, sharply demarcated areas of (i) essentially lost staining for LFB (demyelinated WMLs) or (ii) uniformly thin myelin sheathing (in relationship to axon diameter), which occurs either throughout the WML (fully remyelinated “shadow” plaque) or at the edge of an otherwise demyelinated WML (partially remyelinated plaque) (Prineas et al., 2002; Barkhof et al., 2003; Brück et al., 2003). The minimum area of remyelination required for a WML to be considered partially remyelinated was 10% of the whole WML (Schmierer et al., 2004a). Lesion stage was categorized as either early active (EA: inflammation throughout the WML), chronic active (CA: hypocellular center, inflammation only at the rim of the WML), or chronic inactive (CI: no inflammation) (Fig. 1) (Trapp et al., 1998; van der Valk and de Groot, 2000).

Axon counts were estimated on Bielschowsky-stained slides, by a stereological method using a 21 bar (bar length: 13 μm) quadrate grid graticule (size: 160 × 160 μm^2^) and a final magnification of × 1250. Random points were superimposed over WMLs and surrounding NAWM. On each slide the total number of bars intersecting axons was counted in 12–16 areas of both the WML and surrounding NAWM (Brück et al., 1997). The counts were then averaged for each WML and respective NAWM ROI.

Myelin content was quantified in WML and NAWM by assessing transmittance (Tr), defined as the transmitted light divided by the incident light, on LFB-stained sections using a Leica Q500MC digital image analyzer with a 256 grey scale resolution (Leica Cambridge Ltd., UK), which was mounted on a Zeiss photomicroscope 3 (Carl Zeiss, Jena, Germany). The program was set in RGB mode, the white level was kept constant at 75% of the maximum, and a final magnification of × 125 was used. Within every ROI (i.e., a WML or an equally sized region of NAWM adjacent to a WML) the light intensity was assessed in three to five random areas using a field size of 400 × 385 μm. The values obtained from each area were averaged, and then divided by the light intensity transmitted through the object slide (away from the tissue section) to result in the Tr_myelin_ values for WMLs and NAWM (Mize et al., 1988; Gentleman et al., 1999; Schmierer et al., 2004a, b). A high value of Tr_myelin_ reflects low myelin content.

Gliosis in WMLs and NAWM was classified on GFAP-stained slides (i) by visual inspection as mild, moderate, or severe, and (ii) quantitatively in WMLs and surrounding NAWM by light transmittance in the same manner as for myelin content and expressed as Tr_gliosis_. A low value of Tr_gliosis_ reflects more severe gliosis than a high Tr_gliosis_.

The thickness of LFB- and GFAP-stained sections was assessed using a stereological microscope and a final magnification of × 787.5, in order to control for possible effect of section thickness on the measurements of Tr. On each slide, three to five measurements were performed and averaged.

### Statistical methods

Of the majority of MS cases used for this study more than one tissue block was included in the analysis. Tissue blocks could therefore not be analyzed as independent observations, i.e., without taking into account within-patient dependency. We addressed this “data hierarchy” by analyzing correlation either (a) between patients, (b) within patients or (c) a combination of (a) and (b) taking into account lack of independence.

Two methods were used to compare means: (i) to compare means of variables in WMLs versus NAWM there was insufficient within-patient variability. Hence, between-patient comparisons are being reported in that paired *t*-tests were carried out on patient means averaged across their respective tissue sample. (ii) To compare means between demyelinated and remyelinated WMLs (dWMLs and rWMLs, respectively) it was possible to use a combination of between- and within-subject information. The underestimated standard error and *p* values that would have resulted using a simple *t*-test were inflated by using linear regression according to the Huber–White method (Huber, 1967) for inflating standard error, which resulted in correctly estimated *p* values. The same approach was not possible to test for correlation between MRI and pathology indices, as there was insufficient between-patient variability to sustain consistent use of between-patient information. Therefore, within-patient correlation was obtained using Pearson correlation coefficients estimated from fixed patient intercept regression (Baltagi, 1995), which ignores between-patient differences and fits an estimated average regression slope to each patient's sample (the within-patient correlation measures the strength of the correlation in tissue samples belonging to the same patient).

Potential confounding by slide thickness or batch was investigated by adding these terms to regression models where appropriate (for slide batch, potential confounding is not applicable to within-subject results, all of which were in the same batch). Variation with batch was assessed using a random intercept model to allow for within- and between-patient variability, with restricted maximum likelihood estimation. Where there was doubt concerning normality of model residuals, estimates were checked against those from a non-parametric bias-corrected bootstrap with 1000 replicates (Carpenter and Bithall, 2000), and validity was confirmed. Analyses were carried out using Stata 9.0 (Stata Corporation, College Station, TX, USA).

## Results

The clinical course had been secondary progressive in ten subjects and primary progressive in two. In four subjects, the clinical course could not be determined (Table 1). The estimated mean EDSS was 8 (SD: 1; median: 8.5; range: 6.5–9). The mean brain weight was 1131 g (SD: 113 g, range: 1000–1310 g). The pH of the CSF was 6.9 (SD: 0.4, range: 6.5–7.5). The mean temperature of the tissue during MRI was 22.3 °C (SD: 2.6 °C, range: 17.5–25.1 °C).

### Lesion findings

Seventy-five regions of high signal (RHS) thought to be MS WMLs were detected on *T*_2_w MRI. Twenty-five/75 RHS had to be discarded from further analysis due to (i) poor in-plane co-registration between MRI and specimen (6), (ii) poor identification of different tissue components on histology (5), (iii) misidentification of a vascular lesion (1) or NAWM (10) as WMLs, and (iv) other technical problems (3).

**Fig. 2:**
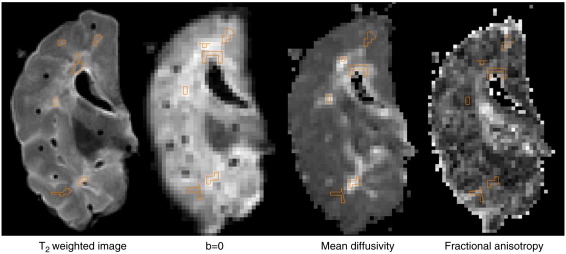
*T*_2_-weighted (*T*_2_w) MRI, *b* = 0, mean diffusivity (MD) and fractional anisotropy (FA) maps of *post mortem* multiple sclerosis brain. On *T*_2_w MRI of a coronal brain slice, seven exemplary regions of interest (ROI; marked in orange) were identified as either normal-appearing white matter (NAWM) or white matter lesions (WMLs). ROIs were visually matched with respective ROI on *b* = 0 images, and then co-registered to the MD and FA maps. One of the five WMLs seen on *T*_2_w MRI (asterisk) could not be detected on diffusion maps.

Of the remaining 50 WMLs, a further 12 had to be discarded because no clear correspondence could be determined between the *T*_2_w MRI and *b* = 0 map (Fig. 2). Hence, 38 histologically confirmed WML, visible on *T*_2_w MRI as well as *b* = 0 images were studied. Between one and six WMLs in each brain slice (mean 2.4 WMLs; SD: 1.4 WMLs) could be used for the analysis. Quantitative MR values were also obtained in two to three regions of NAWM in each slice and then averaged.

Twenty-eight/38 WMLs (73.7%) were demyelinated, three were fully remyelinated (7.9%) and seven partially remyelinated (18.4%) (Table 1). Two/38 (2.6%) WMLs were classified as early active, nine (23.7%) as CA, and 27 (71.1%) as CI WMLs. All (fully or partially) rWMLs were CI WMLs.

Twenty-two/38 WMLs (57.9%) were hypo-intense and 16/38 (42.1%) iso-intense on *T*_1_w MRI. Fully rWMLs (shadow plaques) represented two/22 (9.1%) *T*_1_ hypo-intense WMLs and 1/16 (6.25%) *T*_1_ iso-intense WMLs. Partially remyelinated WMLs contributed three/22 (13.6%) *T*_1_ hypo-intense WMLs and four/16 (25%) *T*_1_ iso-intense WMLs.

Fibrillary gliosis was classified in 35/38 WMLs and observed as being moderate in five/35 (14.3%), and severe in 30/35 (85.7%) WMLs.

### Comparison of MS lesions and NAWM

MD, FA, myelin content (Tr_myelin_), axonal count and gliosis (Tr_gliosis_) all differed significantly between WMLs and NAWM. Results were similar when the analysis was restricted to (partially or fully) rWMLs versus NAWM: rWMLs displayed significantly higher MD, more pronounced gliosis and lower FA, myelin content and axonal count (Table 2).

### Correlation of MR diffusion and pathology

**Fig. 3:**
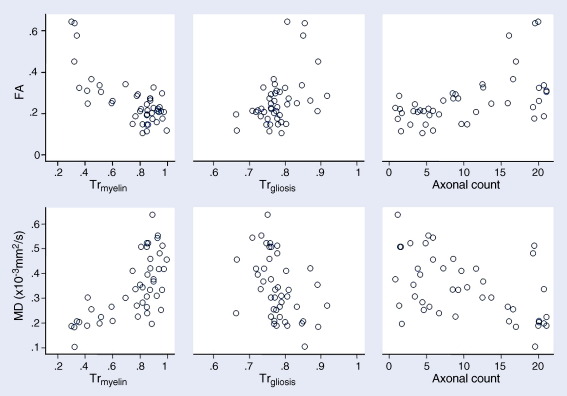
Correlation between diffusion indices and quantitative histology in *post mortem* multiple sclerosis brain (white matter lesions, normal-appearing white matter). The plots illustrate the association of fractional anisotropy (FA) and mean diffusivity (MD) with (i) transmittance of sections stained for Luxol fast blue (Tr_myelin_, inversely proportional to myelin content), (ii) transmittance of sections immuno-stained for glial fibrillary acidic protein (Tr_gliosis_, inversely proportional to severity of gliosis) and (iii) axonal count. See Table 3 for correlation coefficients.

Myelin content (Tr_myelin_) correlated with FA (*r* = − 0.79, *p* < 0.001) and MD (*r* = 0.68, *p* < 0.001). A similarly strong correlation was detected between axonal count and both FA (*r* = 0.70, *p* < 0.001) and MD (*r* = − 0.66, *p* < 0.001). A weaker correlation emerged between both MD and gliosis (*r* = − 0.55, *p* = 0.002) and FA and gliosis(*r* = 0.50, *p* = 0.004) (Table 3, Fig. 3).

### Correlation between neuropathological features

**Fig. 4:**
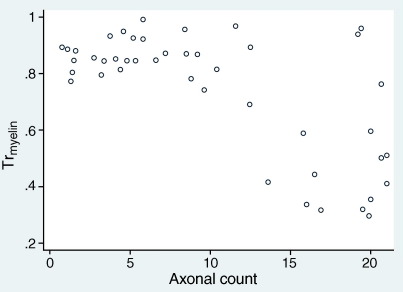
Correlation between transmittance (Tr) of sections stained for Luxol fast blue (Tr_myelin_, inversely proportional to myelin content) and axonal count in *post mortem* multiple sclerosis brain (white matter lesions, normal-appearing white matter).

Myelin content (Tr_myelin_) was strongly associated with axonal count (*r* = − 0.81, *p* < 0.001) (Table 2, Fig. 4). Both Tr_myelin_ and axonal count were moderately associated with Tr_gliosis_. More severe gliosis correlated with lower myelin content and a smaller axonal count (Table 3).

### Regression analysis of confounding correlations

#### Predicting myelin content

The univariate correlation of Tr_myelin_ with FA (− 0.79, *p* < 0.001) is reduced to partial correlation − 0.65, *p* < 0.001 after adjustment for MD. The univariate correlation between Tr_myelin_ and MD (*r* = 0.68, *p* < 0.001) is reduced to partial correlation 0.43, *p* = 0.019 after adjusting for FA.

These results suggest that FA and MD are independently associated with Tr_myelin_. The correlations remain robust when adjusting for section thickness of the LFB-stained slides.

#### Predicting axonal count

The univariate correlation between axonal count and FA (0.70, *p* = 0.001) is substantially reduced and no longer significant when adjusted for Tr_myelin_ (partial *r* = 0.16; *p* = 0.399). Albeit reduced, the association between axonal count and FA remains significant after adjustment for Tr_gliosis_ (partial *r* = 0.57, *p* = 0.001).

The univariate correlation between axonal count and MD is substantially reduced and no longer significant after adjustment for Tr_myelin_ (partial *r* = − 0.24; *p* = 0.212). The association between axonal count and MD remains significant after adjustment for Tr_gliosis_ (partial *r* = 0.51, *p* = 0.005).

**Fig. 5:**
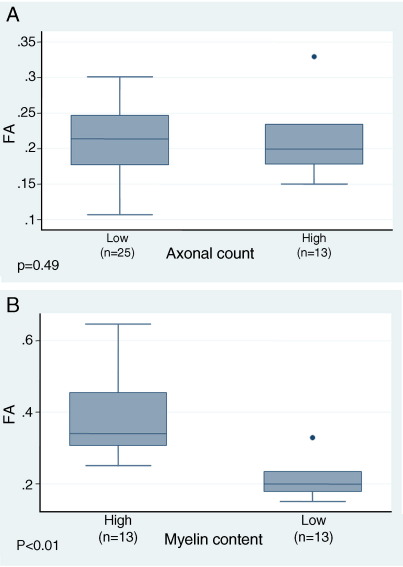
Box-plots of fractional anisotropy (FA) in regions of interest (ROIs) with (A) low myelin content (i.e., high transmittance of sections stained for Luxol fast blue, Tr_myelin_) and (B) high axonal count. ROIs with low myelin content and high axonal count are those below and above the respective means over all ROIs. ROIs with low myelin content are shown divided into those with low and high axonal counts: no difference in FA is evident between these subgroups (*p* = 0.49). ROIs with high axonal counts are shown divided into those with high and low myelin content: ROIs with high myelin content have a higher FA than ROIs with low myelin content (*p* < 0.01).

Together, these results suggest that the correlations of axonal count with both FA and MD are not independent of the (strong) correlation between axonal count and Tr_myelin_, but unrelated to the severity of gliosis. In a post hoc analysis box-plots were produced to further illustrate the relationships between FA, myelin content and axonal count (Fig. 5).

#### Predicting gliosis

We were unable to assess whether the univariate association between Tr_gliosis_ and FA (*r* = 0.50, *p* < 0.01) is independent of Tr_myelin_, as neither association is significant in a model with both. Nor has it been possible to assess whether the association between Tr_gliosis_ and MD (*r* = − 0.55, *p* < 0.01) is independent of Tr_myelin_.

### Comparison of demyelinated and remyelinated lesions

dWMLs had ∼ 60% lower axonal count (*p* = 0.029) and a trend for higher MD (*p* = 0.061) and lower FA (*p* = 0.167) than rWMLs (Table 2).

### Other histopathological lesions subgroups

No significant differences in MD or FA were seen between CA and CI WMLs or between *T*_1_ hypo-intense and iso-intense WMLs. There were too few EA WMLs to investigate this lesion category.

None of the described relationships was substantially affected by estimated EDSS, age, disease duration, time between death and tissue retrieval, or time between death and MRI.

### Batch and section thickness

The mean thickness of the histological sections was 9.4 μm (SD: 3.6 μm), and 4.8 μm (SD: 0.7 μm) for LFB- and GFAP-stained samples, respectively. Staining of the samples for LFB and GFAP was performed in nine and six batches, respectively. Tr_myelin_ in WMLs did not vary with batch, but significant batch variation was seen for Tr_myelin_ in NAWM (*p* < 0.001), and borderline significance variation for Tr_gliosis_ in WMLs and NAWM. None of the observed pathology–MR correlations were affected by including section thickness and batch in the regression analysis (correlation coefficients were altered by no more than ± 0.01, ± 0.05 and significant *p* values by not more than ± 0.001, ± 0.002 when adjusting for batch and thickness, respectively).

## Discussion

### Pathological correlations of the MR measures

This study revealed strong univariate correlation of myelin content as well as axonal content with MD and FA in chronic *post mortem* MS brain. Multivariate analysis of our data, however, suggests that both indices MD and FA are primarily affected by myelin content, whereas their correlation with axonal count is largely explained by the strong association of the latter with myelin content. This finding challenges the concept that an increase in MD and decrease in FA of WMLs and/or NAWM necessarily indicate the presence of axonal loss (Hesseltine et al., 2006) in patients with progressive MS: on the contrary, the findings suggest that loss of myelin *per se* is sufficient to produce a significant change in both of these diffusion measures. Caution is therefore recommended in using either MD or FA to make inferences on axonal integrity *per se*.

Both, MD and FA as well as myelin content, axonal count and the severity of gliosis were clearly different between WMLs and NAWM. These differences were also robust in the subgroup of rWMLs versus NAWM, though only marginal evidence emerged for a difference of MD (and even less so for FA) between rWMLs and dWMLs. This may be due to the limited sample size: whereas in the analysis of rWMLs versus NAWM all rWMLs could be included (of all cases in which rWMLs were detected areas of NAWM were available for comparison), only cases with both lesion types were included in the analysis of rWMLs versus dWMLs. Compared to WMLs the standard deviation of FA was larger in NAWM; FA in the former may have been consistently low due to most WMLs exhibiting complete demyelination, whereas in the NAWM, FA may vary considerably depending on the orientation of fiber pathways and bundles in the ROI.

The extent of fibrillary gliosis correlated moderately with higher MD and lower FA. This correlation, however, was not independent of the correlation of MD and FA with myelin content (and axonal count). Hence, our data suggest that in chronic *post mortem* MS brain MD and FA appear to be mainly affected by a loss of structure due to demyelination and – to a lesser degree axonal loss – resulting in larger intercellular spaces (Barnes et al., 1991; Larsson et al., 1992; Werring et al., 1999). Histologically, the network of gliotic fibers appears to restrict the disorganization that would result from demyelination and axonal damage alone, and it has been suggested that this “repair” mechanism (fibrillary gliosis) thereby limits the magnitude of MD and FA alterations in MS brain (Rovaris et al., 2005). Our data suggest, however, that the potential to quantify this effect using indices of MR diffusion may be limited, at least in *post mortem* MS brain: in univariate correlation, more severe gliosis was actually correlated with a *higher* MD, although this apparent association is probably secondary to the correlation between gliosis and demyelination.

No difference was detected in this study for any quantitative index (MRI, histology) between CA and CI WMLs (Fig. 1) suggesting that the extent of inflammation in chronic *post mortem* MS brain tissue may have a minor effect on measures of MR diffusion. However, the number of histopathologically active WMLs in this sub-study was small, and the overall sample biased towards the late chronic stage of MS with limited inflammatory activity of WMLs. *In vivo* studies in patients with MS suggested that compared to chronic WMLs, MD is significantly higher and FA lower in acute (Gd-enhancing) WMLs (Werring et al., 1999; Roychowdhury et al., 2000), though published results have been equivocal in this respect (Rovaris et al., 2005). More EA and CA WMLs will have to be sampled to further elucidate the significance of inflammation for MD and FA changes in MS brain.

With cautious reference to *post mortem* changes of diffusivity in autopsy tissue (see below) our study suggests that in patients with MS MD and FA both primarily reflect myelin content, while axonal damage and (to a lesser degree) gliosis may also affect these two measures of DTI. As our sample was limited to chronic *post mortem* MS brain, no conclusions can be drawn about (i) the possible effect of early inflammatory changes and (ii) changes in the NAWM *per se* on MD and FA.

### Methodological aspects of the study

Compared to published values acquired during an *in vivo* study of MS patients using the same MR system (Werring et al., 1999), the present *post mortem* study exhibits reduction in average MD in NAWM of ∼ 75% (0.22 versus 0.88 × 10^− 3^ mm^2^/s) and in WMLs of ∼ 60% (0.35 versus 1.11 × 10^− 3^ mm^2^/s); the reduction in average FA is ∼ one-third in NAWM (0.38 versus 0.56) and ∼ 56% in WMLs (0.22 versus 0.50). These decreases likely reflect several factors, including dehydration of *post mortem* tissue, the lower temperature during *post mortem* MRI, and the breakdown of energy-dependent ion transport mechanisms resulting in a net influx of water into cells—thereby reducing the extra-cellular space (Le et al., 1992; Lee et al., 1998; Gass et al., 2001). The reduction in diffusivity of brain tissue following death is a well-recognized phenomenon. In cats the diffusion coefficient compared to *pre mortem* values dropped by up to 10% within 3 min and by up to 50% within 15 min following death (Moseley et al., 1991). A similar decrease of the diffusion coefficient *post mortem* has been reported in rats (Wheeler-Kingshott et al., 2000). Due to ethical and logistical issues involved in the handling of human *post mortem* brain (Bö et al., 2004) it is difficult to systematically establish, in human brain specimens, early *post mortem* changes (i.e., within the first few hours after death) *per se* of MR diffusion. In our sample, including time between death and MRI as a covariate in the analysis revealed no systematic drift of the acquired data. Nevertheless, the effects of temperature, *post mortem* time and tissue decay need to be kept in mind when trying to infer likely *in vivo* changes from data obtained from *post mortem* tissue. It is noteworthy that in a study of mouse brain diffusion anisotropy indices remained virtually unchanged when images were acquired immediately after death (Sun et al., 2003), and minimizing the *post mortem* time should provide a closer approximation to the *in vivo* state. Nevertheless, the differences in MD and FA that are seen between WMLs and NAWM *in vivo* (higher MD and lower FA in WMLs) were clearly retained in this *post mortem* study; the observations therefore allow inferences to be drawn concerning the pathological substrates of diffusion abnormalities in MS *in vivo*.

In order to take account of the reduced diffusivity in *post mortem* brain samples and to improve the quality of DTI maps, the MR acquisition had to be optimized differently from an *in vivo* protocol. Susceptibility artefacts were reduced by introducing a multi-shot (instead of a single-shot) EPI protocol, and a *b* factor of 1940s/mm^2^ (i.e., almost twice as that used in optimized *in vivo* protocols), was chosen to accommodate for the reduced diffusivity in *post mortem* tissue. These measures allowed the acquisition of images and maps (Fig. 2) of reasonable quality. An even greater *b* factor of ∼ 4000s/mm^2^ would be desirable to further increase the sensitivity to white matter changes at low FA values (Jones et al., 1999).

Throughout this study we have focused on the investigation of MS white matter. In our experience it is sometimes difficult to unequivocally distinguish in *post mortem* brain between grey and white matter, particularly when the distinction between the cortex and white matter is concerned. This may, at least in part, be due to volume averaging (Moore, 2003). The use of an inversion recovery experiment may help to achieve better contrast between grey and white matter in *post mortem* brain (Geurts et al., 2005). However, this MR modality has not been applied in the current study.

Compared to scanning brain slices, MRI of the whole *post mortem* brain (Newcombe et al., 1991) may have some advantages, including better three-dimensional anatomic relationships and landmarks, less dehydration and – if scanned *in situ* – fewer susceptibility artefacts due to retained CSF (Bö et al., 2004). Moreover, the brain *in situ* may decay more slowly due to the “sterile” encapsulation of the brain in the skull, though microbial contamination is only one among several factors (including *post mortem* time, temperature and humidity) with influence on tissue degradation. Apart from the limited availability of whole brains for individual research projects, considerably more logistical efforts, time and resources are required to set up a “whole-corpse” imaging protocol (Bergers et al., 2002), and it may be difficult to achieve optimum correspondence between the MR scans and their pathological correlates (Bö et al., 2004), though results from studies using marmosets are encouraging (Hart et al., 1998).

### Future areas for MRI–pathology study in MS

Other quantitative MR measures that have been investigated previously using *post mortem* MS brain include MT ratio (van Waesberghe et al., 1999; Barkhof et al., 2003; Schmierer et al., 2004a), *T*_1_ hypo-intensity (van Walderveen et al., 1998; Barkhof et al., 2003; Schmierer et al., 2004a), *T*_1_-RT (Schmierer et al., 2004a) and *T*_2_-RT (Moore et al., 2000). All have been associated with myelin in MS brain with evidence for MT ratio being closely related (Barkhof et al., 2003; Schmierer et al., 2004a). The reliability of *post mortem* observations of MT ratio and *T*_1_-RT has been enhanced by their apparent stability for up to 36 h *post mortem* compared to published *in vivo* data (Schmierer et al., 2004a). With FA and MD also appearing to reflect myelin content and – to some degree – axonal loss, further research is needed to seek more specific quantitative MR measures of both axonal loss and myelin content *per se*. Since axonal loss is the likely pathological substrate of irreversible and progressive disability in MS – whereas demyelination alone has potential to allow functional and structural recovery (through restoration of nerve conduction and remyelination, respectively (Smith et al., 2006)) – there is a pressing need for a more specific axonal marker.

Recent studies using animal models suggested that measures of directional diffusivity other than FA may be able to distinguish between axonal degeneration (increased axial diffusivity; normal radial diffusivity) (Song et al., 2003) and loss of myelin *per se*, at least in the early phase of myelin breakdown (increased radial diffusivity; normal axial diffusivity) (Song et al., 2002). These studies used longitudinally aligned structures such as the optic nerve (Song et al., 2003), the corpus callosum (Song et al., 2005; Sun et al., 2006) and the spinal cord (Kim et al., 2006). It may be worthwhile to also investigate the radial and axial diffusivities of these and similar structures in *post mortem* MS brain.

Further research should also investigate the pathological correlates of MR tractography obtained from DTI data sets in *post mortem* MS brain tissue. MR tractography is a relatively novel application of DTI, which may have potential in quantifying the degree of nerve fiber damage and loss in MS (Ge et al., 2005; Lin et al., 2005) and other conditions such as amyotrophic lateral sclerosis (Ciccarelli et al., 2006). Longitudinal brain structures including the optic nerve, spinal cord or corpus callosum may be most appropriate to investigate the quantitative pathological correlates of MR tractography in MS brain.

## Conclusion

In a *post mortem* study of subjects with progressive MS and chronic WMLs, we have found a strong correlation of two standard diffusion measures – MD and FA – with myelin content and – to a lesser degree – axonal count. MD and FA appear in the present study to be useful indicators of demyelination in MS. Further work might usefully apply a similar multivariate analysis approach – as in the present study – to investigate these and other potential MR markers for their specificity in quantifying both myelin content and axonal count.

## Figures and Tables

**Table 1:** Overview of patients and lesion characteristics in *post mortem* brain slices of 16 patients with multiple sclerosis (38 white matter lesions, WMLs)

Course	*n* patients	*n* white matter lesions							
*T*_2_w	*T*_1_w [percent of *T*_2_w]	dWMLs	rWMLs	Partially rWMLs	EA	CA	CI
SP	10	24	12 [50]	16	3	5	0	5	19
PP	2	4	3 [75]	3	0	1	2	0	2
Unclear	4	10	7 [70]	9	0	1	0	4	6

SP = secondary progressive MS; PP = primary progressive MS; *T*_2_w = *T*_2_ weighted; *T*_1_w = *T*_1_ weighted; dWMLs = demyelinated WMLs; rWMLs = remyelinated WMLs; EA = early active; CA = chronic active; CI = chronic inactive.

**Table 2:** Comparison (means, standard deviations, *p* values) of (i) white matter lesions (WMLs) versus normal-appearing white matter (NAWM), (ii) remyelinated WMLs (rWMLs) versus NAWM and (iii) demyelinated WMLs (dWMLs) versus rWMLs in *post mortem* brain of patients with multiple sclerosis

	WMLs (*n* = 38)	NAWM (*n* = 16)	*p*	rWMLs (*n* = 7)^a^	NAWM (*n* = 2)^a^	*p*	dWMLs (*n* = 5)	rWMLs^b^ (*n* = 3)	*p*
MD	0.35 (0.09)	0.22 (0.04)	< 0.01	0.31 (0.07)	0.22 (0.03)	0.01	0.51 (0.12)	0.33 (0.10)	0.06
FA	0.22 (0.06)	0.38 (0.13)	0.01	0.23 (0.07)	0.38 (0.16)	0.01	0.19 (0.05)	0.24 (0.08)	0.17
Tr_myelin_	0.85 (0.09)	0.45 (0.10)	< 0.01	0.77 (0.07)	0.43 (0.12)	< 0.01	0.87 (0.04)	0.80 (0.06)	< 0.01
Axonal count	8.3 (5.1)	18.7 (2.0)	< 0.01	11.3 (4.8)	18.9 (2.5)	0.01	3.5 (1.7)	9.2 (0.4)	0.03
Tr_gliosis_	0.76 (0.04)	0.80 (0.03)	0.08	0.75 (0.05)	0.80 (0.02)	0.05	0.76 (0.02)	0.79 (0.03)	0.59

MD = mean diffusivity [× 10^− 3^ mm^2^/s], FA = fractional anisotropy; Tr_myelin_ = transmittance of slides stained for Luxol fast blue; Tr_gliosis_ = transmittance of slides immuno-stained for glial fibrillary acidic protein.

a rWMLs were detected in two cases only.

b All fully remyelinated (i.e., shadow plaques).

**Table 3:** Correlations^a^ between indices assessed in *post mortem* brain of patients with multiple sclerosis

	Pearson r* (*n* regions/*n* patients)			
MD	FA	Tr_myelin_	Axonal count
FA	− 0.62 (51/16)			
Tr_myelin_	0.68 (45/15)	− 0.79 (45/15)		
Axonal count	− 0.66 (44/15)	0.70 (44/15)	− 0.81 (44/15)	
Tr_gliosis_	− 0.55 (45/15)	0.50 (45/15)	− 0.51 (45/15)	0.62 (44/15)

MD = mean diffusivity; FA = fractional anisotropy; Tr_myelin_ = transmittance of slides stained for Luxol fast blue; Tr_gliosis_ = transmittance of slides immuno-stained for glial fibrillary acidic protein.

a Within-patient Pearson correlation coefficients.

* *p* Value of all correlations was <0.01
