# Supplementary material for: Diffusion tensor imaging of post mortem multiple sclerosis brain
Source: Neuroimage. 2007 Apr 1;35(2):467–77. doi: 10.1016/j.neuroimage.2006.12.010 (PMC1892244; doi:10.1016/j.neuroimage.2006.12.010)

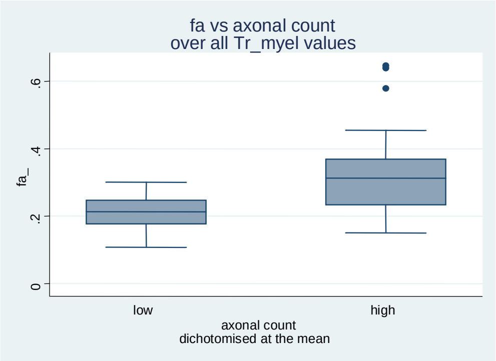

[LEFT]When Tr_myelin values are ignored, fa appears to take a higher value in the high compared to the low axonal count strata, an apparent positive association, as reflected in the scatter plot when axonal count is continuous rather than dichotomised.

[BOTTOM RIGHT]When the comparison is repeated only in the high Tr_myelin stratum, the difference in fa between axonal count strata disappears: ie, if Tr_myelin is held constant, fa does not vary with axonal count. In other words, after adjusting for Tr_myelin, the positive fa vs aconal count association disappears.

[BOTTOM LEFT] When the comparison is repeated only in the low Tr_myelin stratum, there are only high values of the axonal count, consistent with the negative axonal count Tr_myelin relationship.

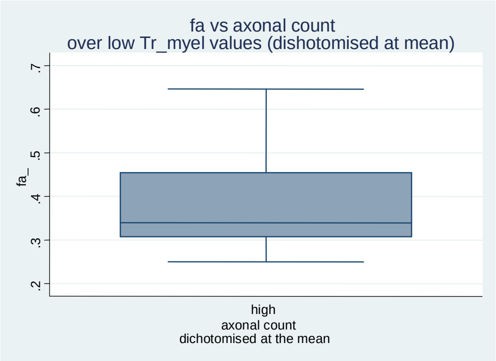

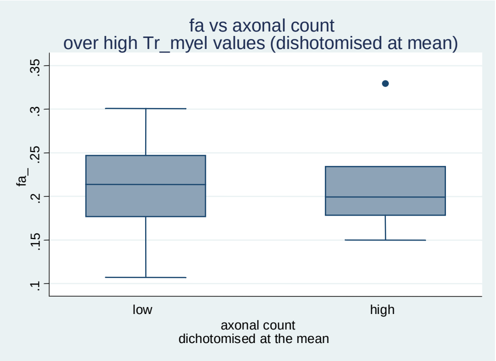

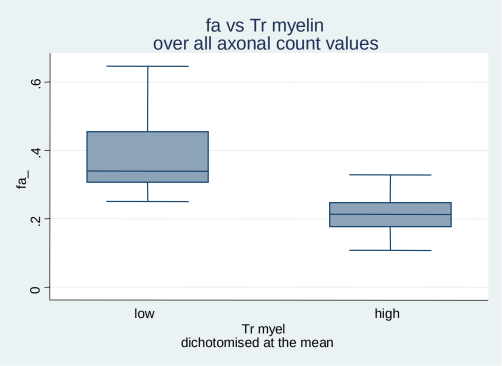

[LEFT]When axonal count values are ignored, fa appears to take a higher value in the low compared to the high Tr_myelin strata: an apparent negative association.

[BOTTOM RIGHT]When the comparison is repeated only in the high axonal count stratum, the difference in fa between Tr_myelin strata **remains**: ie, if axonal count is held constant, fa **still** varies with Tr_myelin, the negative association remains. This is the important asymmetry in the triangular relationship which the multiple regression detects.

[BOTTOM LEFT] When the comparison is repeated only in the low axonal count stratum, there are only high values of Tr_myelin, consistent with the negative axonal count Tr_myelin relationship.

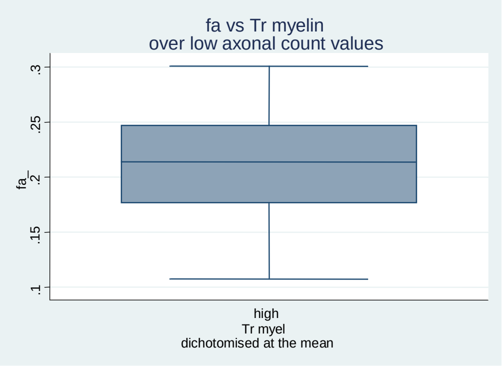

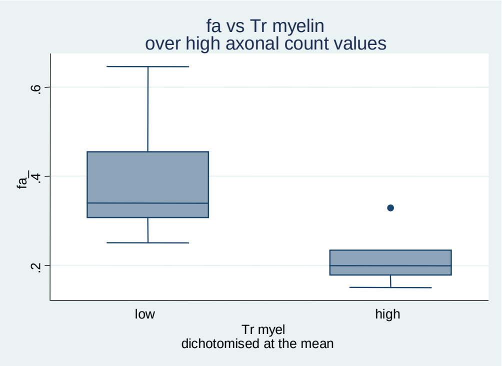

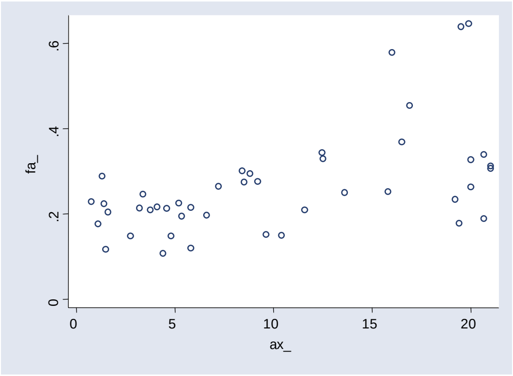

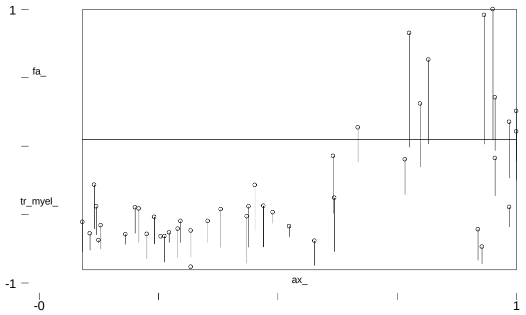

The same point can be shown with the continuous scatters: above we have fa vs ax, ignoring Tr_myelin. The TOP RIGHT scatter is in fact a projection of the 3D graph BOTTOM RIGHT (where the top right graph has been turned anticlockwise by 45 degrees), in which the depth, representing Tr_myelin, is ignored.

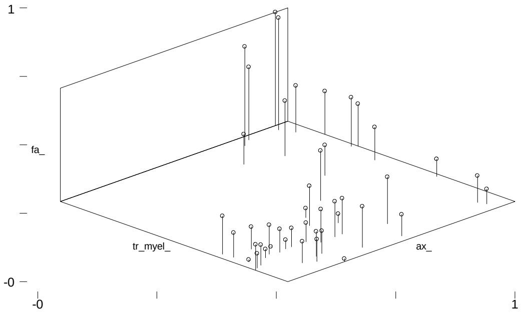

When the Tr_myelin (depth) is taken into account, the high fa & axonal count values, giving the illusion of a positive association, are in fact deeper in the graph, and the fa vs ax relationship is induced by the lower Tr_myelin values of this graph region.

The full range of axonal count values is only present in the high Tr myelin stratum (the graph foreground), and in this stratum the fa vs axonal count relationship disappears.

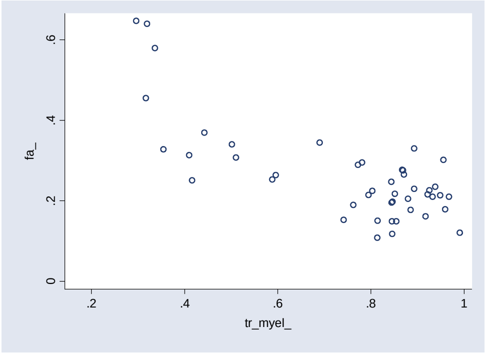

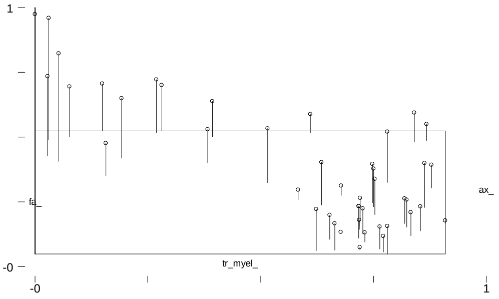

The full range of Tr_myelin values occurs only in the high ax stratum, but there the fa vs tr_myelin negative association remains, in contrast to the fa vs axonal association.

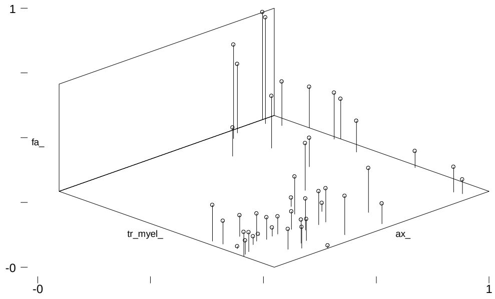

And note also that the axonal vs Tr_myelin (negative) association, represented by the ‘floor’ of the 3Dgraph, occurs among both ‘tall’ (high fa) and ‘short’ (low fa) points.

Here are the dichotomised graphs to show this.

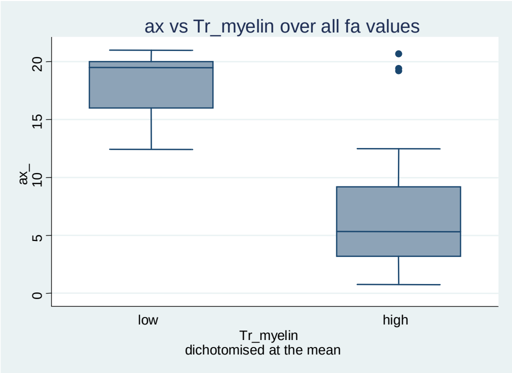

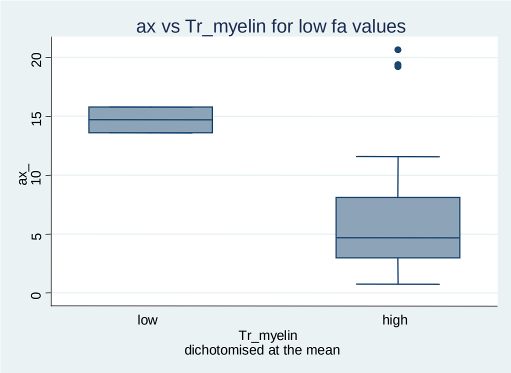

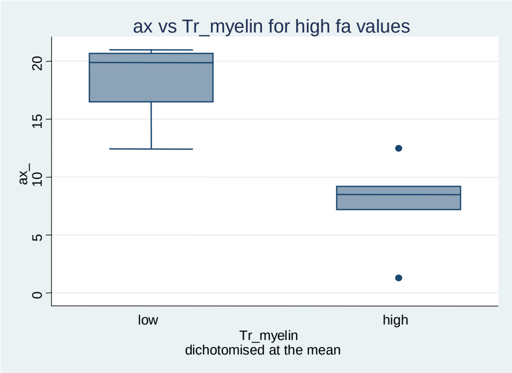

Supplement: Supplementary file 1 [file mmc1.doc]
